# Review of 11 national policies for rare diseases in the context of key patient needs

**DOI:** 10.1186/s13023-017-0618-0

**Published:** 2017-03-31

**Authors:** Safiyya Dharssi, Durhane Wong-Rieger, Matthew Harold, Sharon Terry

**Affiliations:** 1 Pfizer Inc., 235 E 42nd St, New York, NY 10017 USA; 2 Canadian Organization for Rare Disorders, 151 Bloor Street West, Suite 600, Toronto, ON M5S 1S4 Canada; 3 Genetic Alliance, 4301 Connecticut Ave., NW, Suite 404, Washington, DC 20008-2369 USA

**Keywords:** National rare disease plan, Policy, Legislation, Patient advocacy, Europe, Asia, North America, South America

## Abstract

Rare diseases collectively exert a global public health burden in the severity of their manifestations and the total number of people they afflict. For many patients, considerable barriers exist in terms of access to appropriate care, delayed diagnosis and limited or non-existing treatment options. Motivated by these challenges, the rare disease patient community has played a critical role, elevating the patient voice and mobilizing legislation to support the development of programs that address the needs of patients with rare diseases.

The US Orphan Drug Act of 1983 served as a key milestone in this journey, providing a roadmap for other countries to introduce and implement similar orphan drug legislation; more recently, the European Union (EU) has gone further to encourage the widespread adoption and implementation of rare disease plans or strategies designed to more adequately address the comprehensive needs of patients with rare diseases. Despite these legislative efforts and the growing contributions of patient advocacy groups in moving forward implementation and adoption of rare disease programs, gaps still exist across the policy landscape for several countries. To gain deeper insights into the challenges and opportunities to address key needs of rare disease patients, it is critical to define the current status of rare disease legislation and policy across a geographically and economically diverse selection of countries. We analyzed the rare disease policy landscape across 11 countries: Germany, France, the United Kingdom, Canada, Bulgaria, Turkey, Argentina, Mexico, Brazil, China, and Taiwan. The status and implementation of policy was evaluated for each country in the context of key patient needs across 5 dimensions: improving coordination of care, diagnostic resources, access to treatments, patient awareness and support, and promoting innovative research. Our findings highlight the continuing role of the patient community in driving the establishment and adoption of legislation and programs to improve rare disease care. Further, we found that while national rare disease plans provide important guidance for improving care, implementation of plans is uneven across countries. More research is needed to demonstrate the effect of specific elements of rare disease plans on patient outcomes.

## Background

### Rare disease is a global public health issue

Individual rare diseases by definition affect few people, but cumulatively have a major impact on public health. While the definition of rare disease varies by country, prevalence-based definitions range from 1 in 500,000 to 1 in 2000 [1, 2]. In the United States, the Orphan Drug Act of 1983 defined a rare disease by the number of affected people; by this definition, the actual prevalence of rare diseases varies with population numbers [3]. A disease is considered rare if it affects fewer than 200,000 individuals, whereas other countries define a rare disease based on prevalence rates [1, 3–6]. For example, in Europe, diseases are considered rare when they affect fewer than 5 individuals in 10,000 [7]. In Brazil, the definition is similar to the World Health Organization definition, as those affecting less than 65 out of 100,000 individuals [8]. In Taiwan, a rare disease is defined as a disease that is prevalent in fewer than 1 in 10,000 individuals [9].

An estimated 5000 to 8000 rare diseases have been identified worldwide, affecting approximately 6 to 8% of the population [10]. While rare diseases exhibit considerable diversity in etiology and clinical presentation, most are severely disabling with serious effects on life expectancy and physical and mental abilities [11]. Further, rare diseases constitute a major economic burden independent of a country’s size and demographics; these costs arise from increased healthcare spending and lost productivity [1, 12–14].

Many rare disease patients experience barriers in access to care, and fewer than 10% receive disease-specific treatment [15]. Delayed diagnoses, limited access to resources, and absence of specific therapies often preclude patients from receiving proper, timely care. Even brief delays in diagnosis may have profound effects on outcomes; for over 40% of rare disease patients, treatment delays are precipitated by misdiagnoses [11, 16]. When patients are diagnosed, many are unable to access resources such as centers of expertise, coordinated care, patient support systems, and effective treatment. For many rare diseases, there are no effective treatments and information on disease progression is limited. Therefore, research into the natural history and underlying pathophysiological mechanisms of rare diseases is necessary to develop a foundation for discovering targeted medicines.

The patient community plays a critical role toward addressing these challenges, by elevating the patient voice and partnering in the development of programs to address the needs of patients with rare diseases. Approximately three and a half decades ago, the patient movement to create awareness and support for rare diseases took shape in the United States under the banner of the National Organization for Rare Disorders. Joint efforts under the leadership of a mother (Abby Myers), a congressman (Henry Waxman), and an actor (Jack Klugman) garnered the national stage in a series of headline events that captured the public imagination and mobilized Congress to pass the world’s first orphan drug legislation in 1983. This was a seminal event in the development of national rare disease policy and transformed the landscape for rare disease drug development; from 10 new drugs in the decade preceding the Orphan Drug Act to over 500 new drugs in the succeeding three decades [3, 17]. The success of the Orphan Drug Act informed efforts to introduce orphan drug legislation in other countries [18] such as Japan, Singapore, South Korea, Australia, and the European Union.

While these legislative efforts were more focused on the development of innovative treatments for rare disease patients, the European Union went one step further, broadly ensuring that comprehensive policy programs addressed all needs of rare disease patients. In 2009, the European Council adopted the “Recommendation on an Action in the Feld of Rare Diseases,” which supports the adoption of national plans and strategies for responding to rare diseases. The council recommended the development of national plans or strategies to address comprehensive needs, including diagnosis, treatment, care, and support of citizens with rare diseases [10]. Countries were encouraged to consider in these plans aspects such as the improvement in the awareness of rare diseases, the support for research into rare diseases, the development of centers of expertise, the empowerment of patient organizations, and the implementation of a robust healthcare infrastructure.

A number of countries preceded the European Recommendation and France was already ahead of other member states, adopting the First National Plan for Rare Diseases in 2004 [19]. The plan created centers of expertise that were responsible for coordinating diagnosis and provision of health and social care, writing national protocols for diagnosis and care, collecting data, and conducting clinical trials. France’s National Plan served as the impetus and model for all European Union countries, which have developed or are in the process of developing National Plans or Strategies for rare diseases [19]. In the Asia Pacific region, Taiwan had a set of established programs, where rare diseases are included in the National Disability Registry and government social services programs for patients with rare diseases that include health-related subsidies and tax deductions, school fee reductions, employment supports, and transportation discounts. Similarly, Japan had enacted special legislation in 1972 to address “nanbyo,” or rare and intractable diseases, with about 130 “tokutei shikkan” or special designated rare diseases receiving public subsidized care [20].

As national plans for rare diseases evolve, patients and patient advocates continue to play an integral role in moving forward the implementation and adoption of the programs outlined in the plan. The European Project for Rare Diseases National Plans Development (EUROPLAN) was a project co-funded by the EU Commission to promote and implement National Plans or Strategies to tackle rare diseases, to share relevant experiences within countries, linking national efforts with a common strategy at a European level [21]. Importantly, EUROPLAN formally included organized patient input and has played a key role within the European Union in guiding the establishment and implementation of National Plans or Strategies for rare diseases [21]. To this end, EUROPLAN included a specific role for the European Organization for Rare Diseases a European umbrella group of non-governmental associations of patients with rare diseases. The European Organization for Rare Diseases ensured that EUROPLAN processes were reflective of patient viewpoints, in part by organizing a series of conferences to discuss coordination of the European Union and national policy [16]. A committee of rare disease experts, the European Union Committee of Experts on Rare Diseases (EUCERD) Joint Action, was set up by the European Commission to formulate and implement policy to combat rare diseases [22, 23]. The mandate of EUCERD Joint Action ended in July 2013 and been replaced by the EC Expert Group on Rare Diseases in 2013 [24]. The EC Expert Group continues to have a strong presence of patients’ organizations in the field of rare diseases. The elaboration of policies in the field of rare diseases and improvement of the codification of rare diseases has been continued through the scope of RD-Action, a Joint Action co-funded via the 3rd EU Health Programme for the years 2015–2018. The European Organization for Rare Diseases (EURORDIS) continues to play an integral role representing the voice of patients and driving forward the adoption of important rare disease and orphan medicine legislations at the European level. In addition, similar organizations at a national level, such as the Canadian Organization for Rare Disorders (CORD), actively play an instrumental role in driving forward policy development. Both organizations have developed key position statements that outline top priorities that public decision makers should consider when addressing the needs of rare disease patients. While each countries policies and programs reflect the priorities of their health care system and needs of rare disease patients, the EURODIS and CORD position statements provide a general, but comprehensive framework that could be applicable to a majority of countries. As such, in this paper, we analyzed the rare disease legislation and associated policies, regulations, and programs across a diverse sample of 11 countries according to the goals outlined in the European Organization for Rare Diseases and Canadian Organization for Rare Disorders position statements. The objective of the review is to understand where there are opportunities for further policy development by examining how these policies and programs might align with key needs of the rare disease community, which are summarized in five dimensions: 1. improving coordination of care, 2. diagnostic resources, 3. access to treatments, 4. patient awareness and support, and 5. promoting innovative research. We also considered the contributions of patient advocacy groups and included some external measures of healthcare provision as an initial consideration for how current rare disease policy might align with the healthcare climate. The findings from this study will inform future studies evaluating how specific elements for National Plans or Strategies for rare diseases translate to provision of care for rare disease patients.

## Methods

Information for this study was collected and analyzed from 11 countries: Germany, France, the United Kingdom (UK), Canada, Bulgaria, Turkey, Argentina, Mexico, Brazil, China, and Taiwan. Countries included in the analysis were selected to be geographically and socioeconomically diverse, and represent a wide range of rare disease policy development. The US was excluded since it lacks a formal National Rare Disease Plan and policy variation across states confounds comparisons with other countries. The term National Rare Disease Plan (NRDP) was used to refer to strategies and plans that have been developed to support a comprehensive and integrated approach to the delivery of health and social care for rare disease patients.

NRDPs were assessed using publically available official documents and secondary research. Based on guidance published [25] for rare disease policy, the following elements of NRDPs were evaluated for each country:

- National policy
- Access to treatment
- Diagnosis programs
- Coordination of care
- Research
- Patient engagement

The data were then assessed on the basis of our five keys needs of the rare disease community: coordination of care, diagnosis, access to treatments, patient awareness and support, and research.

## Results

The status of National Rare Disease Plan (NRDP) policy elements for each of the 11 countries is presented in Table 1. Countries were arranged in the table according to overall healthcare/economic status, defined by gross national income (GNI) per capita and healthcare spending (relative to gross domestic product [GDP]), and the level of NRDP development. France and Germany have well-developed NRDPs that are being implemented at the national level. With the highest healthcare spending and average wealth healthcare ranks of the 11 countries surveyed, France and Germany have centralized national funding, good access to treatments, robust research initiatives, coordinated networks, and cross-border collaborations in place for rare diseases. The UK and Canada have similar healthcare structures and NRDPs that are both in the implementation stage with a number of robust programs in place. Bulgaria and Turkey have draft or full plans approved and comparable levels of healthcare spending, but their plans have had limited implementation. Argentina, Mexico, and Brazil have similar healthcare spending relative to gross domestic product (GDP) but NRDPs are awaiting development or are very early in implementation. Finally, the NRDPs of China and Taiwan are not proposed or are proposed but not fully implemented.

**Table 1:** Summary of rare disease policy and regulatory frameworks across 11 countries

	France	Germany	UK	Canada	Bulgaria	Turkey	Argentina	Mexico	Brazil	China	Taiwan
National Policy	Two plans implemented; Launch of third plan underway	Adopted; implementation underway	Plan adopted for all 4 UK nations; implementation underway	Adopted; implementation underway	Approved; limited implementation	Draft guideline passed; implementation delayed	Approved; awaiting implementation	No uniform plan	In early implementation stage	None	Adopted; implementation underway
Authorization Process	Central EU authorization COMP OD Designation	Central EU authorization COMP OD Designation	Central EU authorization COMP OD Designation	Accelerated review available	Central EU authorization COMP OD Designation	No defined OD designation	ANMAT regulates conditions for drug approval Streamlined authorization available for US or EU-approved drugs OD registration process in place	Accelerated review available Process in place for OD classification	Accelerated review available; timelines vary No special registration process for ODs	Delays in OD drug approval despite fast-track program No distinction between ODs and regular drugs	Accelerated review available Process in place for OD classification
Early Access Programs	Processes in place for ODs with and without market authorization	Granted during the third phase of the clinical trial and when the product’s safety and efficacy are guaranteed.	Early Access to Medicines scheme approved, but not specific to RD	No plan for early access; Patients may apply to the Special Access Program in special circumstances	No legal provision for early access	Program available for patients with unmet medical needs meeting defined criteria	Program for free access to drugs from other countries in place	No programs in place	No legislation or programs in place Unregistered medicines can be imported upon request	ODs yet to be authorized may be available through donation programs	No formal NP in place, but policies implemented Unregistered medicines can be imported upon request
Current Access to Treatment	Market authorization required ODs can only be prescribed in RD competence centers (+500 in France) No OD-specific funding	Several initiatives exist to improve access to ODs No OD-specific funding	NICE HST review process for ultra-orphan drugs Scotland’s New Medicine Fund allocated £80 M for ODs	ODs assessed through same HTA process as other drugs Provincial Drug Plans-special provisions for ODs	Access to ODs improved markedly over last 4 years Special considerations for HTAs of ODs 10% of annual NHIF budget allocated to ODs	Pathways for access are defined No OD-specific funding	Comprehensive RD patient care: Act 26.689 not yet implemented Refund system provides financial support to Social Health Service for select drugs	Limited information available No OD-specific funding	Limited access through public health system No OD-specific funding	No special access considerations No OD-specific funding	Special access considerations Several drugs approved recently Separate budget not covered by NHI
Diagnosis Programs	Neonatal screening available for 4 pathologies (2 RDs) Inequality in access to tests	Newborn screening available for 14 conditions Screening and genetic testing mandatory, but no specific RD policies	The UK Rare Disease Strategy does not specifically contain early diagnosis programs Expansion of RD testing in newborns as of 2015	Provincial newborn screening programs vary in number and conditions Health Ministers to implement screening programs for 22 conditions	National program for early diagnosis expected to be fully funded/operational by 2016	Few public diagnosis centers Neonatal screening for 3 RDs through Ministry of Health Genetic testing at universities; testing abroad possible	Legislation passed, but no programs in place	No early diagnosis initiatives Limited newborn screening Early Diagnosis Clinic under development	Newborn screening available for 6 RDs Main rare diseases research centers is located in the Federal University of Rio Grande do Sul,for diagnosis	No national screening and/or diagnostic programs available	Improved screening and diagnosis in development Nation-wide newborn screening program for 26+ diseases
Coordination of Care	131 centers of reference and 501 centers of competence are in place	RD expert centers and networks for cross-border research in place	UK Strategy for Rare Diseases Calls for specialist centres to deliver coordinated care, 150 providers of highly specialized services	Centers of Expertise in specific rare diseases; some linked to research across sites with adult clinics No national coordinating body for all RD Centers of Expertise	Not in place RD committee not yet operational	Few centers with specialized services; mostly university hospitals and research centers	Planned: Act 26.689 Cibersalud launched to strengthen specialist networks “Hiptour” patient-lead initiative provides medical guidance to HCPs	No developed centers, but functionally active network of 12 civil organizations Lysosomal Storage Disease Network: diagnosis, treatment, support	National policy initiated with accreditation of specialized health centers in progress;	No comprehensive specialist centers Centers for specific RDs located in Beijing and Shanghai Most mature treatments for hematologic RDs	No comprehensive specialist centers 10+ RD genetic counseling centers approved Some NPs implemented but coordinated care not fully developed
Research	300+ funded clinical research projects Funding allocated for basic and clinical research Banque Nationale de Données Maladies Rares national database launched European/international collaborations developed	28 RD organizations supported by Ministry of Education and Research BMBF funding 12 research projects; €23 million allocated for 3 years Cross-border research projects No central coordinated RD registry	Collaborative initiatives with pharmaceutical companies for patient-centered research exist UK10K project on RD genetics Public Health England intends to build national RD registry	Canadian Institutes for Health Research funds basic and translational research Proposed formal inclusion of RD research in CIHR, Genome Canada, and IRDiRC	Growing awareness but few research initiatives in place Centralized patient registry approved but not yet operational	No initiatives, networks, or cross-border collaborations No national registries, but Turkey participates in European registries Comprehensive national epidemiological survey underway	No national initiatives in place Some research conducted with support from patient organizations, research grants, or private initiatives Legislation passed, but registries still under development	No funded research projects or initiatives Development of RD patient database in progress	Few incentives; no long-standing initiatives; Initiative in 2014 by the National Council of Scientific and Technological Development (CNPq), which screened and funded 15 research projects devoted to rare diseases (diagnosis and treatment) Bill to secure RD research funding under review No national registries Patient groups collect data DORA program and FEMEXER RD database in development	CARDPT national research program initiated in 2013 Cross-border initiatives gaining momentum No national centralized patient database/registry Registry launched at local level in 2013 Patient group-developed database in progress	No RD-specific national registry National disability registry in place and hemophilia registry in development
Patient Engagement	Established patient organizations are engaged and play a role in RD policy	Established patient organizations are engaged and play a role in RD policy	Established patient organizations play active role Stakeholder engagement by Rare Disease UK and Specialized Healthcare Alliance	Patient group support and advocacy facilitated by CORD National Strategy launched with Rare Alliance Canada	Patient organizations actively engaged Implemented support and education programs and played a role in the launch of 13 RD registries	Little information available	Patient organizations play active role Collaborations with government aided implementation of RD legislation and programs	Patient organizations play an important, active role	Patient organizations play an important role	Patient advocacy groups limited but localized RD groups raise awareness/promote legislation Chinese Organization for Rare Disorders active in RDI	Strong patient support/advocacy groups active in RD policy TFRD helped pass 2000 Rare Disease Control and ODA
Gross National Income per Capita (2015)	40,580	45,790	43,340	47,500	7220	9950	13,640	9710	9850	7820	22,723
Healthcare Spending (% GDP)	11.5	11.3	9.1	10.4	8.4	5.4	4.8	6.3	8.3	5.5	6.6
Average Wealth Healthcare Rank	1.8	2.3	2.3	2.8	7.8	6.5	7.0	7.3	6.5	5.0	8.8

*Abbreviations* Administración Nacional de Medicamentos, Alimentos y Tecnología Médica; *BMBF Bundesministerium für Bildung und Forschung*, Federal Ministry of Education and Research; *CARDPT* China Alliance for Rare Disease Prevention and Treatment; *CIHR* Canadian Institutes of Health Research; *CORD* Canadian Organization for Rare Disorders; *EU* European Union; *DORA Doenças Raras*, rare diseases; *FEMEXER Federación Mexicana de Enfermedades Raras*, Mexican Federation for Rare Disorders; *HTA* Health technology assessment; *IRDiRC* International Rare Diseases Research Consortium; *NHI* National Health Insurance; *NHIF* National Health Insurance Fund; *NICE* National Institute for Health and Care Excellence; *NP* national plan; *OD* orphan drug; *RD* rare disease; *RDI* Rare Diseases International; *TFRD* Taiwan Foundation for Rare Disorders

The patient organization actions for each country are presented within each of the five dimensions representing key needs of the rare disease community, summarized below.

### Dimension 1: Coordination of care

The term “coordination of care” was used to describe resources designed to improve the provision of timely, equitable, and evidence-informed care. Programs and terminology vary by country but include Centers of Expertise (COEs) on rare diseases, integrated healthcare administrative databases, and national registries for rare diseases. While some countries attempt to coordinate their approach to rare disease management using centers of expertise, many countries do not, either because they have not yet adopted this approach or are employing different strategies.

The concept of dedicated and comprehensive centers for rare diseases was originally developed by France for its first National Rare Disease Plan (NRDP), and then adopted by the EU upon the request for national plans nearly a decade ago. France has over 600 centers that coordinate research, train healthcare professionals (HCPs), and facilitate diagnoses (Table 1) [26]. Although centers of expertise do not exist, per se, in the United Kingdom (UK), the UK Strategy for Rare Diseases recommended the creation of specialist centers to deliver coordinated and expert care required to support the needs of rare disease patients [27]. The Birmingham Centre for Rare Diseases is an example of the way one hospital has created a specialist center for treating patients with rare diseases. In addition, the English National Health Service lists about 150 providers of highly specialized services, some of which are directed at rare diseases, such as liver transplant services, enzyme replacement therapy, and proton beam therapy for specific cancer treatments [28]. Further, the European Union recently issued criteria for designating European Reference Networks (ERNs) across the European Union for treating rare medical conditions and in December 2016 approved the first wave of 23 European Reference Networks (ERN)s [27, 29].

In the Asian countries surveyed, no formal NRDPs for Centers of Expertise are in place. The Taiwanese Bureau of Health Promotion has approved medical institutions to serve as centers for diagnosing and treating rare diseases, and Taiwan has at least 10 approved genetic counseling centers [30]. However, the coordinated care across centers is not fully developed. Given the relatively small geographic size of Taiwan, patients may not have to travel far to access treatment, but, in larger countries, dispersal of uncoordinated resources is a challenge. To this point, barriers in the coordination of care in Latin American countries are due to large population sizes spread across vast landscapes, with many patients located far from urban centers where centralized expertise tends to be located. However, to support access to expertise in rural areas, many countries are employing technology to address this issue. In Argentina, the Health Ministry recently launched Cibersalud, an innovative tool to strengthen the network of specialists in the country and facilitate referrals, diagnosis, and monitoring. Cibersalud seeks to promote consultations among healthcare professionals from different establishments in the country and to assist teaching activities, through the provision of technological equipment and the development of applications that allow videoconferencing between establishments. More recently, the Ministry of Health launched an interactive course on Rare Diseases aimed at strengthening the diagnosis of rare diseases. The course is intended for healthcare professionals, including pediatrics, family medicine and general practitioner residents of public hospitals and training is conducted through on online discussion on clinical cases and biweekly telemedicine meetings (i.e. Cibersalud) [31, 32].

In countries without formal centers for rare diseases, patient groups are playing an active role in supporting coordinated care. For example, in Argentina, the Pituitary Diseases Association implemented “Hiptour” to provide medical guidance to professionals for the diagnosis of pituitary diseases in new patients. In Mexico, the Lysosomal Storage Disease Patient network has helped to create a model of comprehensive care starting with diagnosis and including treatment and support that has garnered high-level political attention and endorsement. However, it is centrally located and also limited in outreach resources. Overall, identifying and creating Centers of Expertise or comparable programs represent an important step toward consolidating existing rare disease expertise.

### Dimension 2: Diagnosis

An accurate and timely diagnosis is predicated on availability of universal or highly accessible screening and diagnostic programs and services. Across the surveyed countries, policies and practices varied significantly. Neonatal screening has the potential to contribute to an early diagnosis and management of a fraction of rare diseases when there is an effective intervention which can avoid or mitigate severe consequences and/or death if provided early enough Multiple conditions can be identified from a single bloodspot collected at birth. However it is important to note that the composition of a newborn screening panel can vary between regions, depending on local prevalence. A number of newborn screening programs commonly utilize criteria established by JMG Wilson and F. Jungner in 1968 to decide whether a particular condition is a suitable candidate for screening [33]. A recent report on the practices of newborn screening implemented in EU member states noted that most jurisdictions also take guidelines of scientific societies in consideration [34]. However, many aspects are subjective and there is not always agreement about which disorders should be part of the panel. With the advent of new diagnostic technologies, debates have currently arisen as to how screening programs should adapt.

Many countries have implemented core programs of neonatal screening (eg, Taiwan, Brazil, UK, Germany). Taiwan has implemented a nation-wide newborn screening program that covers at least 26 diseases [30]. Similarly, Germany tests for 14 conditions nationwide [35]. While this number is far removed from the total number of identified rare diseases, only a limited number of neonatal screening assays are currently available. In 2016, Canada announced that the Health Ministers had agreed to a list of 22 core conditions for newborn screening programs across all provincial and territorial jurisdictions, although some provinces were already routinely testing for 30+ diseases [36]. These requisites are all considerably lower than the number of rare diseases that could be tested with a single bloodspot, and this is true of most countries globally (except for the United States, where some states test for 50 or more diseases). In most of the countries surveyed, there was a distinct lack of formal programs for newborn screening and/or infrastructure/resources to support implementation, and this was apparent across geographic regions, with Argentina, Brazil, China, and Taiwan as leading examples where other rare disease initiatives are in place or in planning but limited attention has been given to core newborn screening as an essential component of the rare disease program. For example, in Brazil despite the progressive translation of genetic research into a clinical field and efforts by the Ministry of Health to establish the National Newborn Screening Program (Programa Nacional de Triagem Neonatal – PNTN) in 2001, progress still remains to take place. Currently, only six diseases are included in the formal newborn screening program [8, 37]. The panel still is modest when compared with the screening programs of other countries, including Taiwan with 26 diseases and Germany with 15 diseases.

Beyond the neonatal stage, diagnosis of rare diseases can be even more difficult, resulting in long delays in diagnosis and many misdiagnoses. Geographical challenges exacerbate this problem since long journeys to visit specialists in different regions can impair accurate diagnosis. In Mexico, some patients describe “medical pilgrimages” to different doctors so as to reach a diagnosis, which can take 5 years or longer to achieve. A 2012 European Organization for Rare Diseases survey of eight rare diseases representing 12,000 patients found that 25% of patients had to wait between 5 and 30 years for a diagnosis, 40% received an initial erroneous diagnosis (leading to wrong medical interventions), and 25% had to travel to a different region to obtain a diagnosis [38]. Similarly, a survey conducted by the Canadian Organization for Rare Disorders found that about 20% of patients waited between 6 and 14 years to get a diagnosis and 60% consulted 3 to 20+ specialists on the way to a diagnosis. Importantly, the availability of robust diagnosis and neonatal screening programs can significantly reduce the patient diagnostic odyssey and bridge the gap between disease onset and diagnosis that, for many countries, represents a major barrier in access to care.

Beyond awareness, an important strategy that is being promoted in many countries is education for health care practitioners to develop and expand expertise in rare diseases. For example, the Argentinian Health Ministry launched a tool to promote collaboration between professionals from different institutions across the country with the goal of improving diagnosis. In many emerging healthcare systems, patient groups have taken an active role in promoting professional education and collaboration. The mobilization of a cystic fibrosis patient group was important in shaping decisions on what diseases are screened in Brazil, while, in Mexico, patient groups have spurred the development of early diagnosis funding, patient registries, and efforts to raising rare disease awareness. Progress in these directions has also occurred in the Asia-Pacific region, as the Taiwan Foundation for Rare Disorders (TFRD) coordinates efforts with the Bureau of Health Promotion to send specimens for testing in both foreign and domestic labs for improving diagnosis and subsidizing costs for rare disease testing at local hospitals.

### Dimension 3: Access to treatments

The availability of rare disease treatments is addressed in a number of national regulatory and legislative policies designed to facilitate orphan drug designation, authorization, and early access programs. The European Union passed regulation (EC) No 141/2000, on orphan medicinal products in 2000, which covers all of the countries in the EU, including the UK, Germany, Bulgaria and France represented in this survey [39]. Further, the Committee for Orphan Medicinal Products (COMP) was established with the responsibility to recommend orphan designation of medicines for rare diseases to the European Commission for final decision [40]. In the European Union under the Regulation on Orphan Medicinal Products, medicines designated as orphan medicinal products are provided incentives, including protocol assistance. More recently, under the new Priority Medicines (PRIME) scheme, the European Medicines Agency (EMA) will offer early and enhanced scientific and regulatory support and enable accelerated assessment for therapies that address significant unmet medical need and offer the potential to bring a major therapeutic advantage to patients [41]. Several other countries have expedited review processes for orphan medicines, namely, Turkey, Mexico, Brazil, China [42], Taiwan, and Canada [43], but the criteria and process vary across the countries [44, 45]. In Brazil, the Agência Nacional de Vigilância Sanitária (ANVISA), enacted a regulation (Resolution RDC 28) in 2007 that was further clarified in 2008 (RDC 16), stating that orphan drugs should receive priority review and a decision on approval within 75 days [8]. In practice, the approval can take much longer and regulatory developments are underway to improve review timelines. In Mexico, despite no specific regulation for expedited approval, orphan medicinal products that have been approved by other Agencies, such as the US Food and Drug Administration or the European Medicines Agency, can be expedited via a letter of recognition, and this mechanism has shortened the authorization timeline to approximately 6 months [45]. Argentina has had similar success with a program administered by the Argentinian National Administration of Medicines, Food, and Medical Technology that streamlines authorization requirements for orphan drugs approved in the United States or the EU [45]. In Canada, drugs for unmet needs or urgent situations are eligible for expedited review.

For approved drugs, several countries have policies in place to ensure timely patient access to treatment following regulatory approval. The UK’s National Institutes for Health and Clinical Excellence (NICE) have established a separate review process for “Highly Specialised Therapies” which address ultra-orphan diseases (population of less than 1 in 10,000) [46]. Elsewhere, within the UK, The Scottish Medicines Consortium has also developed a new approach for reviewing rare disease medicines designed to increase the influence of patients and clinicians in the appraisal process. This “Patient and Clinician Engagement group” can be convened to address additional benefits that may not be represented in the conventional clinical and economic assessment process. Scotland also has a New Medicine Fund that was recently expanded to £80 million a year allocated to orphan drugs to ensure patient access to the most advanced therapies for diseases with unmet needs [47]. Similarly, in Bulgaria, special considerations are made for health technology assessments for rare disease treatments [48]. Further, Argentina employs a unique system of refund that administers financial support to the Social Health Service for certain drugs.

### Dimension 4: Patient awareness and support

To what degree do patients and families get the education and support for their rare disease? For each country, we looked at three key indicators: public awareness directed toward better identification and support, patient education toward, among other things, engagement, advocacy and access to resources, and healthcare professional development to enhance diagnosis, treatment, and care. The countries within our sample varied considerably regarding who was involved, what was done, and how much was achieved. Public awareness ranged from comprehensive campaigns organized by dedicated national or international organizations to local awareness initiatives implemented by stand-alone groups. In all types of activities at all levels, patient organizations were actively engaged. In the United Kingdom, France, and Germany, established patient advocacy organizations, both disease-specific and cross-disease, organized and delivered a range of programs including education and awareness conferences, patient guides to rare disease research [49], and advocacy that spanned from support for legislative acts to receptions with government leaders [50]. In other countries where few other stakeholders were active, strong national rare disease patient networks had the strongest influence on awareness, policy, and health system response. Examples come from disparate regions in our sample. For instance, in Bulgaria, patient organizations implemented award-winning programs and support for rare diseases and, as importantly, educated and motivated the rare disease patient community not only to better access and navigate the healthcare system but also to advocate for their cause, including a call for legislative action to support their needs and playing an integral role in the launch of 13 epidemiological registries for rare diseases [51]. Similarly, national rare disease collaborations in Argentina and Canada [52] have prompted governments to implement legislation and rare disease programs.

In the Asia-Pacific region where our sample consists of China and Taiwan, rare disease patient support and advocacy groups have been active but the scope and impact of their involvement have varied, reflecting differences in political, economic, geographic, and healthcare contexts. In Taiwan, the non-governmental organization Taiwan Foundation for Rare Disorders (TFRD) deliberately focused on obtaining government support for patients and families with rare diseases. To that end, Taiwan Foundation for Rare Disorders has had a key role in shaping rare disease policy and advocating for rare disease patients. Established nearly two decades ago, the organization leads education, awareness, and advocacy efforts. Through coordinated efforts with governmental organizations, Taiwan Foundation for Rare Disorders helped pass the Rare Disease Control and Orphan Drug Act of 2000. In China, where patient advocacy groups are limited in number, localized and disease-specific, medical rare disease groups have focused their efforts primarily on implementing educational initiatives. Recent efforts have been made to bring together different members of the stakeholder community. In 2011, Chinese medical professionals issued a call to support healthcare, research, drug development, and epidemiological studies for rare diseases [53]. Patient groups, charitable organizations, and pharmaceutical companies have co-hosted patient assistance programs and academic meetings on International Rare Disease Day (February 29) and China Birth Defects Prevention Day (September 12) each year to raise public awareness and promote legislation for rare diseases. More recently, the Chinese Organization for Rare Disorders, formed from an amalgamation of several groups and now representing approximately 40 rare diseases, has conducted several highly innovative and impactful awareness and education programs.

The Chinese and Taiwanese national networks are distinct in their focus and advocacy. Taiwan has more generally focused on within-country awareness raising and lobbying, eschewing most international activities, while the Chinese Organization for Rare Disorders has participated extensively in international arenas and also invited many foreign patient advocates and other experts to support their efforts in China. The differences are due to context. Taiwan is geographically and population-wise a small country, with a relatively strong economy and a high-quality and well-resourced healthcare system that is reasonably accessible to most patients. China, by contrast, is huge in both land mass and population with an emerging economy and evolving healthcare system. The Chinese Organization for Rare Disorders has sought to attract international attention, best practices, and advocacy support. To that end, the Chinese Organization for Rare Disorders is a founding member of the Asia-Pacific Alliance of Rare Disease Organizations and in 2014 was host to the founding meeting of Rare Disease International (RDI), a global alliance of rare disease patients and advocates, to raise awareness, exchange information, and increase the support of the rare disease cause. Participation in the Rare Disease International meeting breaks new ground in that it is an international, patient-led advocacy group and the Chinese Organization for Rare Disorders has the opportunity to host the International Conference on Rare Diseases along with the Rare Disease International meeting in 2017.

Some countries had plans that specifically included health care professional education and training as a key area of focus (Argentina, Germany), while other countries did not emphasize health care professional development (Mexico, China), and this difference reflects, to some degree, the differential involvement of HCPs in the respective patient organizations. The Treatment and Research Centre for Rare Diseases in Germany provides continuing medical education through the German Academy for Further Medical Training on Rare Diseases program, which facilitates interactions and communications between physicians and relevant experts as well as patient organizations [54]. In Brazil, a new National Policy of Integral Attention to People with Rare Diseases includes health care professional training to create specialized teams of professionals to treat patients with rare diseases.

### Dimension 5: Research

Research for rare diseases is commensurate with overall GDP as well as investment in innovation, science, and healthcare; it has been relatively well funded with government investment in some countries (France, Germany, UK, Canada) and not officially supported in other countries (Turkey, Mexico). At a regional level, the European Union has demonstrated a strong commitment to rare disease research through the EU Framework Programme for Research and Innovation. Under the Seventh Framework Programmes for research (2007–2013), over €620 million in support was granted to over 120 collaborative research projects on rare diseases. The funding facilitated the formation of multidisciplinary teams from universities, research organizations, industry, and patient organizations from across Europe and beyond [55]. More recently, Horizon 2020, which runs from 2014 to 2020 continues the European Union’s strong commitment to funding rare disease research [56]. At a country specific level, France, which currently funds over 300 clinical research projects with collaborations across national and international institutions, is seen as a leader in the research space [26]. In Germany, the Federal Ministry of Education and Research (BMBF) is currently funding 12 research consortia since 2012, with more than €23 million for three years and has supported additional funding through initiatives such as the National Genome Research Network [54, 57]. In China, the China Alliance for Rare Disease Prevention and Treatment (CARDPT) has established the first ever national research program of prevention and treatment for rare diseases, which intends to compile basic data on rare disease pathophysiology and natural history, develop and apply medical guidelines, and promote molecular testing for rare diseases [58]. In Canada, the government has committed funding through the Canadian Institutes for Health Research for emerging teams and consortia of researchers, as well as funding to maintain rare disease models and funding for translational research (gene identification, disease registries, and pilot clinics).

In several countries (Bulgaria, Turkey, Argentina, Mexico, Brazil), there appear to be few or no national initiatives to promote research and/or innovation in the treatment of rare diseases. Research projects in Argentina are often conducted and funded through private initiatives, research grants, or support from patient organizations. Similarly, there is no long-standing initiative to promote research on rare diseases at the national level in Brazil; however, a bill intending to secure funding for rare and neglected disease-related research is currently being reviewed by Congress.

Registries are important means of collecting disease, demographic, and treatment data. France is a model for national coordination of registries with their Banque Nationale de Données Maladies Rares, a national organization collecting and organizing data from centers of expertise [59]. French patients enter the registry via the center at which they receive care. In contrast, the UK, Bulgaria, and Argentina, have national patient registries in various stages of planning, but not implemented as of yet. To help support the standardization and sharing of information across rare disease registries, the European Commission, within the EU Program of Community Action in the field of Public Health, has initiated the establishment of a European Platform for Rare Disease Registries to address the challenge of standardizing and sharing information across rare disease registries. [60]. This platform is still in development. Orphanet tracks rare disease registries, databases, and biobanks so as to provide access to this information to all stakeholders

Overall, a movement toward international registries is evolving; for example, national rare disease registries are not well developed in Turkey, but the country has participated in European registries such as TREAT-NMD (for neuromuscular disease) and EUROCARE CF for cystic fibrosis. In Bulgaria, patient groups are working to modify legislation addressing patient registries. In Brazil, no national registries exist for rare diseases, but patient associations have been able to collect information in disease-specific areas. Some countries such as Mexico and China have registries that are geographically dispersed and locally based, but lack standard structures to facilitate patient-specific or whole-population analyses. While a national disability registry exists in Taiwan, registries specific to rare disease are lacking; however, the Taiwan Society of Thrombosis and Hemostasis is currently implementing a patient registry specific to hemophilia.

## Discussion

This study assesses the status of rare diseases in a sample of countries varying across economic, political, healthcare factors and, at the same time, examines the role of patient organizations in shaping national policy and programs, including rare disease legislation, national rare disease plans, and coordinated comprehensive services directed to rare diseases. Most of the countries represented in our sample (France, Germany, the UK, Canada, Bulgaria, Argentina, Brazil, Mexico, and Taiwan) have developed or announced intentions to develop a National Rare Disease Plan, some of which are officially recognized by the government but none, except for France, are experiencing coordinated strategies and policies toward comprehensive implementation. Indeed, the scope and capacity vary considerably. In general, countries that had higher healthcare spending and GNI per capita, as well as national healthcare delivery systems (France and Germany), also had well-developed national plans that encompass early access to treatments, funding for orphan drug access, diagnosis programs, coordinated care, and strong research initiatives. In contrast, the UK and Canada are similar in their financial metrics as well as regionalized healthcare delivery structures, which have challenged the development of national centers of expertise, national guidelines for screening and diagnosis, and national criteria for access to therapy. Scotland, Northern Ireland, and the United Kingdom have unique drug assessment agencies. In the United Kingdom, the National Institute for Healthcare Excellence (NICE) has developed a separate body to assess “highly specialized drugs” primarily for ultra-orphan indications [61], while Scotland introduced a dedicated Rare Conditions Medicines Fund [62] and Northern Ireland considers some form of “ring fenced fund” for orphan drugs [63].

Turkey, Mexico, and China do not have orphan drug legislation; analysis of external financial metrics revealed that these countries also had the lowest GNI per capita and healthcare spending. Countries that were lesser developed and/or had low healthcare spending and GNI per capita were more likely to have National Rare Disease Plans (NRDP) that were not endorsed or implemented. Still, some countries lacking formal NRDPs had legislation/policies/regulations in place to improve access to orphan drug treatment (eg, orphan drug classification and accelerated review policies exist in Mexico).

Clearly, gaps exist between policy and practice. While a number of countries have regulations specific to orphan drugs that are designed to accelerate the authorization process, streamlined processes do not necessarily expedite or guarantee drug approval. In Brazil, the established time frame for responding to authorization requests is 75 days, but authorization times have varied from 13 to 30 months. Bulgaria and Scotland were the only countries across those surveyed to have specific funding allocated to orphan drugs. However, orphan drug licensing in Bulgaria is often delayed from 1 to 6 years.

Despite the lack of formal NRDPs in place, several countries have been able to make great progress in improving processes in the rare disease landscape. Though Canada did not have a national plan in place until September 2015, it has a very strong newborn screening policy and well-established diagnostic centers that exceed what is available in many other countries [36]. In the Asia-Pacific region, Taiwan provides a model for good programs that exist in the absence of specific policy; improved screening and diagnosis programs are in development, led by advocacy groups and funded by the Taiwanese government. Further, Turkey has defined pathways for early treatment, designated specialized centers for coordinated care, and provides screening and diagnosis programs despite delayed implementation of its NRDP.

Several countries, inspired by the progress in Europe, have adapted the consultation process and the core indicators developed by the EUROPLAN project to support the development and implementation of rare disease plans and strategies [64]. In Canada, the Canadian Organization for Rare Disorders played an instrumental role in the strategic development and implementation of the Canadian NRDP, which was distilled from 30+ other NRDPs and asserts goals that are closely aligned with those of the UK Strategy for Rare Diseases [65, 66]. Consistent with the position of the European Organization for Rare Diseases (EURODIS) on rare disease research infrastructure, development, and governance, the inclusion of patients as full and equal partners is a central recommendation set forth by the Canadian Organization for Rare Disorders (CORD) [65, 67].

In other countries, including Mexico, the patient advocacy community is playing a large role in shaping discussions, educating policy makers and driving the political agenda to support national rare disease strategies by actively participating in small working groups with their national Health Commissions. In several countries without formal NRDPs, coordinated and influential disease-specific advocacy groups have provided leadership in addressing gaps and implementing programs to support key needs within the community. One such example of the active role patient groups are playing in supporting coordinated care in the absence of formal centers of expertise is seen in Argentina, where advocates from the Pituitary Diseases Association in Argentina travel around the country and provide medical updates on pituitary diseases to professionals to help identify and diagnose patients. Similarly, patient groups in Bulgaria have been working to modify legislation addressing patient registries to create a centralized registry that is more compatible with international databases. In China, the China Alliance for Rare Disease Prevention and Treatment has established the first ever national research program of prevention and treatment for rare diseases. This demonstrates that despite national prioritization, patient advocacy organizations can drive successful implementation of programs that can help support the key needs of rare disease patients. However, despite the successful adoption of these programs, integrated and coordinated strategies are necessary to ensure holistic access to care and treatment for patients.

To further garner support, patients can further policy development by creating alliances between/among organizations. The Iberoamérican Alliance for Rare Diseases (ALIBER) has established a system across Ibero-American countries to collaborate and share ideas surrounding rare diseases. The recently formed Asia-Pacific Alliance of Rare Disease Organizations (APARDO) represents a collaborative unification of national rare disease groups aimed at improving access to care and treatment of rare diseases in China, Japan, India, Australia, and Singapore. Importantly, many countries analyzed in this study are members of Rare Disease International (RDI), which provides a clear framework for establishing and improving advocacy, awareness, information sharing and networking, research, and partnerships. As the landscape of rare disease policy continues to evolve, the unification of rare disease patient organizations will be essential to driving innovative research with shared resources and best practices, and amplifying the role of the patient voice in the development of programs to address the needs of the rare disease community.

## Conclusion

According to the goals outlined in the EURORDIS and CORD position papers, our study explored the rare disease legislation, associated policies, regulations, and programs across a diverse sample of countries through the perspective of the key needs of the rare disease community. Consistent with previous reports [25, 68, 69], our analysis revealed substantial differences in rare disease infrastructure across countries; however, it was limited in the scope of the countries considered and was not designed to assess the effect of specific policies and structures on patient outcomes. Subsequent analyses should be conducted to correlate policy with the presence of actual programs and, ultimately, their effects on patient care. Importantly, this information will provide a strategic framework that can structure ongoing dialogues within and between countries so as to define best practices in rare disease management and harmonize efforts across the globe in improving patient care.

## Abbreviations

ALIBER: *Alianza Iberoamericana de Enfermedades Raras,* Iberoamérican Alliance for Rare Diseases
ANVISA: *Agência Nacional de Vigilância Sanitária*, National Health Surveillance Agency
APARDO: Asia-Pacific Alliance of Rare Disease Organizations
BMBF: *Bundesministerium für Bildung und Forschung*, Federal Ministry of Education and Research
CARDPT: China Alliance for Rare Disease Prevention and Treatment
COE: Centers of Expertise
COMP: Committee for Orphan Medicinal Products
CORD: Canadian Organization for Rare Disorders
EMA: European Medicines Agency
ERN: European Reference Networks
EU: European Union
EUROPLAN: European Project for Rare Diseases National Plans Development
EURORDIS: European Organization for Rare Diseases
GDP: Gross domestic product
GNI: Gross national income
HTA: Health technology assessment
NICE: National Institute for Health and Care Excellence
NORD: National Organization for Rare Disorders (NORD)
NRDP: National rare disease plans
ODA: Orphan Drug Act
PRIME: Priority Medicines
RDI: Rare Diseases International
TFRD: Taiwan Foundation for Rare Disorders
UK: United Kingdom
